# Absence of mutations in *GJB2* (Connexin-26) gene in an ethnic group of southwest Iran

**DOI:** 10.4103/0971-6866.50863

**Published:** 2009

**Authors:** Hamid Galehdari, Ali Mohammad Foroughmand, Maryam Naderi Soorki, Gholamreza Mohammadian

**Affiliations:** 1 Department of Genetics, Faculty of Science, Chamran University, Ahwaz, Iran; 2 Genetic Consulting Centre, Welfare Organization, Ahwaz, Iran

**Keywords:** Connexin 26, *GJB2*, Iranian Arabs, nonsyndromic autosomal recessive deafness

## Abstract

**BACKGROUND:**

The common *GJB2* gene mutation (35delG) has been previously reported from Iranian patients that were affected with nonsyndromic autosomal recessive deafness. We, therefore, for the first time, investigated the prevalence and frequency of the *GJB2* gene mutation in the Iranian deaf population with Arabian origins.

**MATERIALS AND METHODs:**

We amplified and sequenced the entire coding sequence of the GJB2 gene from 61 deaf patients and 26 control subjects.

**RESULT:**

None of the analyzed samples revealed deafness-associated mutation.

**CONCLUSION:**

This finding differs from several reports from Iran as we have focused on the *GJB2* gene that possesses various mutations as the cause of congenital recessive deafness.

## Introduction

Congenital deafness is a common form of hearing impairment, which occurs in about one in 1000 live births.[1,2] The majority of the affected individuals are ascribed to unknown or presumed genetic factors.

Approximately 80% of the hereditary cases are nonsyndromic and are inherited in an autosomal-recessive mode.[3,4] To date, more than 40 loci have been mapped on human chromosomes that are thought to be linked with the Nonsyndromic Autosomal Recessive Deafness (NSARD), but most of these associated genes in their loci have not yet been identified. One of the main identified causative genes for congenital-recessive deafness is known as *GJB*2, which has been extensively studied in different populations.[5–7]

Up to now, more than 35 different mutations have been described in the *GJB2* gene and most of them are located in the coding region of connexin-26.[8] In several studies, a single guanine deletion at codon 35 (35delG) was identified as a common mutation in the *GJB2* gene.[9–11] However, in other studies, the 35delG mutation was absent or occurred rarely.[8,12–14]

Furthermore, a specific T deletion at codon 167 (167delT) has also been identified in the *GJB2* gene that appears mainly in Ashkenazi Jews.[7]

Therefore, to investigate the prevalence and frequency of the *GJB2* gene mutation, we analyzed NSARD patients with Arabian origin.

## Materials and Methods

### Clinical evaluations of patients

Individuals were ascertained through the genetic counseling center of the welfare organization of Ahwaz. Informed consent was obtained from all family members who participated in the study. Medical and family history and information on pedigree structure were obtained from 61 diagnosed persons with autosomal-recessive nonsyndromic hearing loss and 26 control subjects. Pure-tone audiometry at 2500-8000 Hz was performed for selected subjects. All hearing-impaired family members underwent physical examination. No clinical features, including mental retardation, which would indicate that deafness was part of a syndrome, were observed.

### DNA extraction and polymerase chain reaction (PCR)

From affected and healthy individuals, ethylenediamminetetraacetic acid-treated whole blood was collected and DNA was extracted using the Quick DNA extraction kit (Qiagen, Hilden, Germany) and stored at -20°. Two primers were designed by *primer3out* software from the entire coding sequence of *GJB2* gene (Genbank accesion#M86849) that amplified a fragment of 780-bp length, with the sequences 5′-CTTTTCCAGAGCAAACCGCC-3′ as forward and 5′-TGAGCACGGGTTGCCTCATC-3′ as reverse primer. The amplification was carried out in a thermocycler (Eppendorf AG, Hamburg Germany) for 35 cycles, containing 50 ng genomic DNA, 10 pmol of each primer, 200 μM of deoxy-nucleotide triphosphates (Fermentase Co., Canada), 2.5 μl of 10x PCR buffer, and 2 units of *Taq* polymerase (Roche, Mannheim, Germany) in a volume of 25 μl under the following condition: 30 cycles composed of 40 s at 94°C, 50 s at 64°C, 1 min at 72°C, and 10 min at 72°C after the last cycle. The PCR products were then separated on a 1.5% agarose gel (Sigma Chemical Co., Poole, England) by electrophoresis to check for proper amplification with genomic DNA.

### Direct sequencing of the PCR products

The PCR products were purified by the PCR product extraction kit (Fermentase) and sequenced subsequently with the reverse primer and the Big-Dye Terminator 3.1 Cycle Sequencing Kit by an ABI-PRISM 3700 DNA analyzer (Applied Biosystems, Fairlands, South Africa).

## Results

In the present study, we have investigated a full spectrum of the *GJB2* gene mutations in the Arabian population from southwest Iran by direct sequencing of the PCR products. No GJB2 mutations, including the common 35delG, were found in the analyzed individuals from 61 NSARD patients and their 26 related healthy family members. Simply, one polymorphism (V153I) was detected in two related individuals in the heterozygote mode.

## Discussion

**Figure 1:**
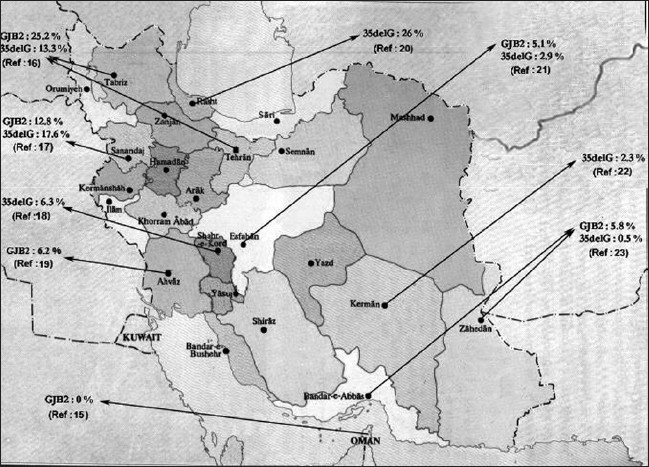
*GJB2* mutation passage through Iran[14]

This is the first report from Iran that shows the total absence of *GJB2* gene mutation in a significant number of NSARD patients with Arabian origins despite *GJB2* gene mutations, which have been described in several populations as well as in the Iran[19] [Figure 1]. These mutations are 35delG, 167delT, and 235delC, which have been found to be very common in Caucasoid, Ashkenazi Jewish, and Oriental populations, respectively.

Carrier frequency of the 35delG mutation in the Caucasoid population occurs 1/31-1/35.[6,24,25] The carrier frequency of the 167delT mutation in the Ashkenazi Jews population is about 4% while the frequency of the 35delG mutation in the same population was reported to be 0.7%.[7] The mutation 235delC was detected in the Japanese population[12,26,27] as the main mutated allele in the deafness patients. The frequency of this mutation in the Japanese population has been determined to be 2/203 by Fuse *et al.*[26] In none of these studies was the 35delG mutation observed. Furthermore, the M34T mutation is also quite frequent in some populations. The carrier frequency for this mutation in the Belgian population, as has been reported by Hilbert *et al.*, was about 2.4%. Carrier screening for the M34T frequency in the Caucasoid populations, as established by Kelley *et al.*[28] and Scott *et al*.,[29] was 3/192 and 1/200, respectively.

The most interesting finding in our study was the absence of any mutation associated with deafness, including the commonly described mutations 35delG, 427C>T(R143W), 167delT, and 235delC in the connexin-26 (*GJB2*) gene in an ethnic group of the Iranian Arab NSARD patients. However, the average mutation rate in the *GJB2* gene has been reported to be about 14.6% in the Iranian population[17,19,21] [Figure 1]. Similar to our finding, the absence of 35delG mutation has also been reported in the Omani population.[15]

In view of the fact that no *GJB2* mutation was identified in our samples, we conclude that the *GJB2* gene is not the major cause of NSARD at least in the Iranian population with Arabian origins. However, it may contribute, by some unknown interactions, with other genes. To date, linkage studies have indicated the presence of more than 40 different genes associated with NSARD. In addition, the Iranian population is composed of several ethnic groups and more work on the basis of the ethnicity is needed to find out which gene(s) is associated with NSARD.

Finally, we presumed that the direct methods to mutation detection could be more confident and those have been compared with other methods as well as the amplification refractory mutation system (ARMS) or the single-strand conformational polymorphism, which may be accompanied with false positive results, as we have had negative experience with the ARMS method (unpublished data) in our laboratory.
